# Ulcers in leprosy patients, an unrecognized clinical manifestation: a report of 8 cases

**DOI:** 10.1186/s12879-019-4639-2

**Published:** 2019-11-29

**Authors:** Denis Miyashiro, Carolina Cardona, Neusa Yuriko Sakai Valente, João Avancini, Gil Benard, Maria Angela Bianconcini Trindade

**Affiliations:** 1 Department of Dermatology, Hospital das Clínicas University of São Paulo Medical School, 255, Dr. Enéas de Carvalho Aguiar Ave, 3rd floor, São Paulo, Brazil; 2 Laboratory of Clinical and Experimental Allergy and Immunology LIM-56, University of São Paulo Medical School, São Paulo, Brazil; 3 Laboratory of Medical Mycology LIM-53, University of São Paulo Medical School, São Paulo, Brazil; 4 Institute of Tropical Medicine of São Paulo, University of São Paulo Medical School, São Paulo, Brazil

**Keywords:** Leprosy, *Mycobacterium leprae*, Skin ulcer

## Abstract

**Background:**

Leprosy is a chronic granulomatous infection caused by *Mycobacterium leprae*. It is a polymorphic disease with a wide range of cutaneous and neural manifestations. Ulcer is not a common feature in leprosy patients, except during reactional states, Lucio’s phenomenon (LP), or secondary to neuropathies.

**Cases presentation:**

We report eight patients with multibacillary leprosy who presented specific skin ulcers as part of their main leprosy manifestation. Ulcers were mostly present on lower limbs (eight patients), followed by the upper limbs (three patients), and the abdomen (one patient). Mean time from onset of skin ulcers to diagnosis of leprosy was 17.4 months: all patients were either misdiagnosed or had delayed diagnosis, with seven of them presenting grade 2 disability by the time of the diagnosis. Reactional states, LP or neuropathy as potential causes of ulcers were ruled out. Biopsy of the ulcer was available in seven patients: histopathology showed mild to moderate lympho-histiocytic infiltrate with vacuolized histiocytes and intact isolated and grouped acid-fast bacilli. Eosinophils, vasculitis, vasculopathy or signs of chronic venous insufficiency were not observed. Skin lesions improved rapidly after multidrug therapy, without any concomitant specific treatment for ulcers.

**Conclusions:**

This series of cases highlights the importance of recognizing ulcers as a specific cutaneous manifestation of leprosy, allowing diagnosis and treatment of the disease, and therefore avoiding development of disabilities and persistence of the transmission chain of *M. leprae*.

## Background

Leprosy is a chronic granulomatous infection caused by *Mycobacterium leprae* that is endemic in some poor resource countries. Brazil is the second country with the highest incidence of leprosy [1]. It is a polymorphic disease with a wide range of neurocutaneous manifestations, which usually appear after a long period of incubation (3–7 years). These manifestations are at least partially determined by the host’s immune response, characterizing leprosy as a spectral disease. Cutaneous lesions of leprosy usually present as hypoesthetic hypochromic macules, papules, plaques, and diffuse infiltration of the skin with alopecia and xerosis [2]. Less frequent manifestations include blisters, hyperpigmented patches, verrucous lesions, macrocheilia, and pure neural leprosy [2–6]. Extra-cutaneous involvement in multibacillary leprosy includes eyes, nasal mucosa, joints, lymph nodes, testicles, liver, and spleen [2]. Diagnosis is based on clinical findings followed by laboratory confirmation, usually the bacteriological examination of slit-skin smears performed by an experienced technician. Current serological assays identify infected individuals but are not able to diagnose all patients with leprosy. In addition, in poor-resource settings, which comprise the endemic areas, diagnosis is in most instances solely based on clinical grounds [2].

Therefore, clinical suspicion after careful examination of skin and neural alterations is of utmost importance for the diagnosis of leprosy. Unfortunately, delayed diagnosis and treatment are still an issue in endemic poor-resource settings and in non-endemic countries due to global migration [7]. Ulcer is not a common feature in leprosy patients, except during reactional states, Lucio’s phenomenon (LP), or secondary to neuropathies [2]. We report eight consecutive leprosy patients, diagnosed between 2010 and 2018 at a single center in São Paulo, Brazil, who had specific cutaneous ulcers that were part of their main leprosy manifestation but were not associated with reactional states, LP or neuropathies,. All patients had delayed diagnosis and seven patients had grade 2 of disability by the time of the diagnosis.

## Cases presentation

**Fig. 1:**
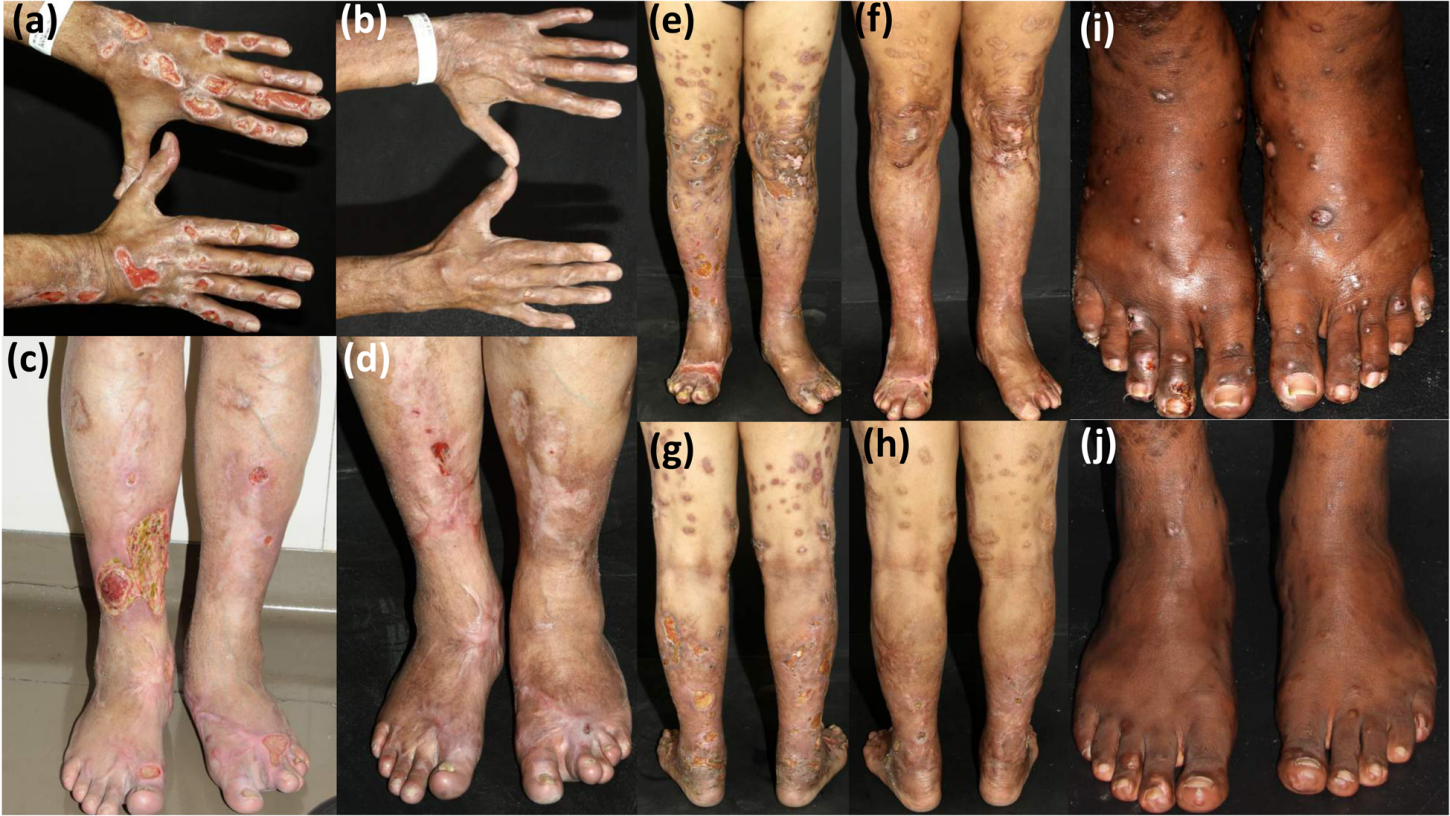
Multiple ulcers on the hands and forearms before (**a**) and after 8 months of MDT-MB (**b**). Multiple ulcers on the legs and feet before (**c**) and after 3 months of MDT-MB (**d**). Multiple ulcers on the lower limbs before (**e** and **g**) and after 12 months of MDT-MB (**f** and **h**). Multiple infiltrated papules, nodules, and ulcerated lesions on the feet in a patients with histoid leprosy before (**i**) and after 7 months of MDT-MB (**j**)

From 2010 to 2018, eight of 411 patients (1.9%) were diagnosed with multibacillary leprosy with ulcers as part of the main leprosy manifestation in our institution. All patients were male. Mean age at diagnosis of leprosy was 47.6 years (range 15.9 to 71.9 years). In all eight patients, leprosy was not associated with reactional states, LP or neuropathy. Ulcers were mostly present on lower limbs (eight patients), followed by the upper limbs (three patients), and the abdomen (one patient) (Fig. 1). Loss of sensation was observed on the lower and upper limbs in six of eight patients; on the back in one patient; and one patient had diffuse loss of sensation. Seven patients had grade 2 disability, and one had grade 1 at diagnosis. Nerve involvement (thickness) comprised: ulnar nerve (six patients), fibular nerve (five patients) and posterior tibial nerve (one patient). All patients had other leprosy associated lesions at diagnosis, as follows: madarosis (five patients), infiltration of ear lobe (four patients), perforation of nasal septae (three patients), oral vegetating and ulcerated lesion on hard palate (two patients), multiple skin-colored infiltrated papules and nodules compatible with histoid leprosy (two patients) [8], foveolar plaques (one patient), and diffuse infiltration with leonine facies (one patient). None of the patients complained of systemic symptoms. Clinical data are summarized in Table 1.

**Table 1:** Clinical findings of the patients with specific skin ulcers caused by leprosy

Case number	Gender	Provenance	Age at diagnosis	Time from the appearance of skin ulcers to the diagnosis of leprosy	Ridley-Jopling classification [9]	Topography of skin ulcers	Type of other skin lesions	Grade of disability at diagnosis	Time to resolution of ulcers after start of MDT-MB
1	Male	Porto União - Santa Catarina	37.2	5 months	BL	Lower and upper limbs, abdomen	Perforation of nasal septae; infiltration of ear lobe	Grade 2	12 months
2	Male	Jacobina - Bahia	15.9	2 years	LL	Hands and feet	Histoid leprosy	Grade 1	7 months
3	Male	Nigeria	34.5	3 years	LL	Lower limbs	Foveolar plaques	Grade 2	6 months
4	Male	Adamantina - São Paulo	71.9	4 years	LL	Lower limbs	Madarosis	Grade 2	7 months
5	Male	Cotia - São Paulo	53.6	1 year	LL	Lower limbs	Perforation of nasal septae; infiltration of ear lobe; madarosis	Grade 2	3 months
6	Male	Adolfo - São Paulo	56.6	2 months	BL	Lower and upper limbs	Perforation of nasal septae; ulcerated vegetating lesion on hard palate; madarosis	Grade 2	8 months
7	Male	Sousa - Paraíba	42.8	9 months	BL	Lower limbs	Diffuse infiltration with leonine facies; histoid leprosy; ulcerated vegetating lesion on hard palate; infiltration of ear lobe; madarosis	Grade 2	3 months
8	Male	Itabirito - Minas Gerais	68.3	3 months	LL	Lower limbs	Infiltration of ear lobe; madarosis	Grade 2	9 months

**Fig. 2:**
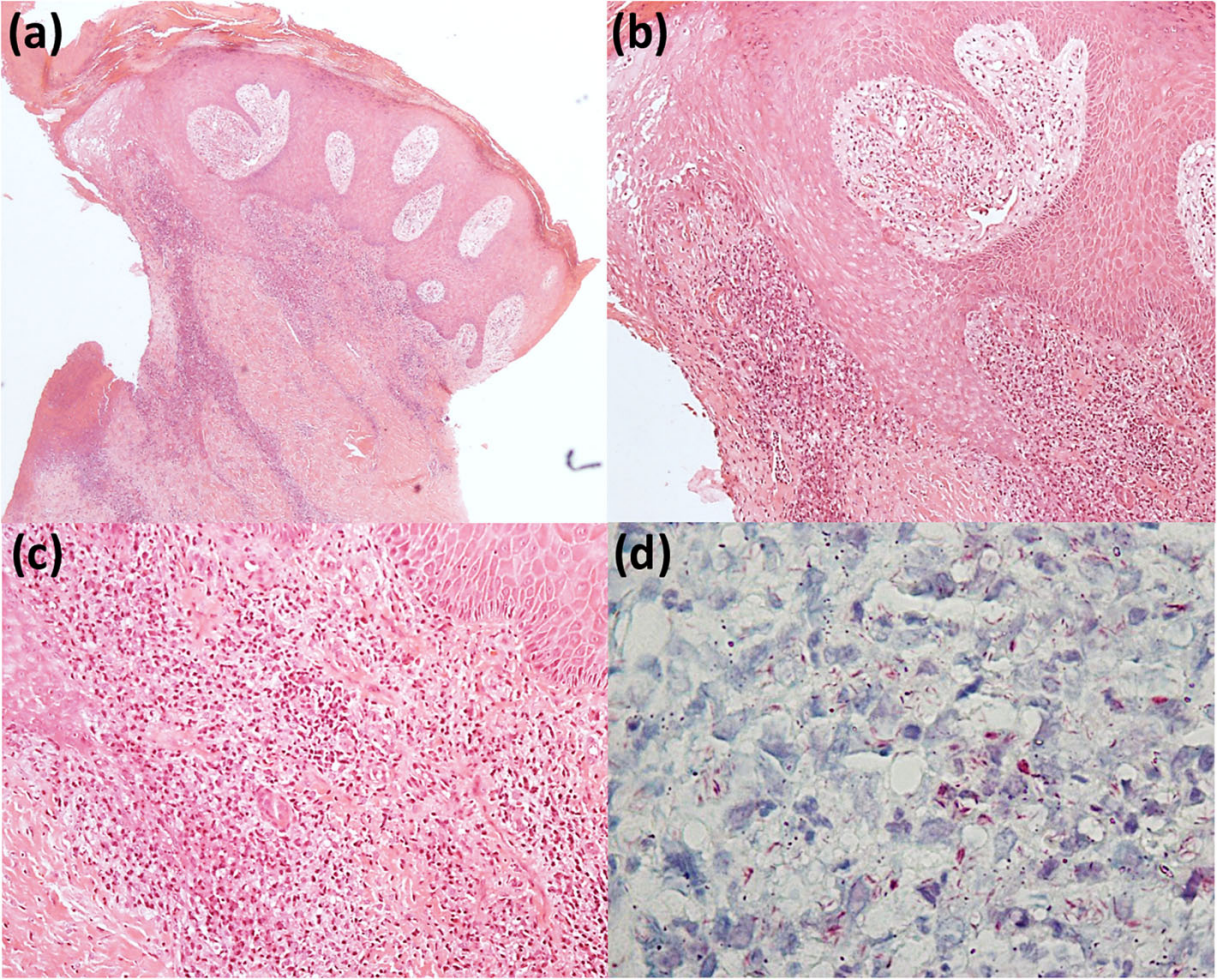
**a** Edge and bed of chronic ulcer: epidermal hyperplasia, dermis with mixed inflammatory infiltrate and fibrosis (40x). **b** Detail of the edge of chronic ulcer: epidermal hyperplasia, vascular proliferation and ectasia, mixed inflammatory infiltrate (100x). **c** Detail of mixed inflammatory infiltrate (lymphocytes, histiocytes, neutrophils, plasma cells) (200x). **d** Fite-Faraco stain with intact isolated and grouped acid-fast bacilli

Mean time from onset of ulcers to diagnosis of leprosy was 17.4 months (2 months to 4 years). Biopsy of the ulcers was performed on seven patients (in patient #2 an infiltrated papule was biopsied): all seven biopsies showed mild to moderate lympho-histiocytic infiltrate with vacuolized histiocytes. Two patients had neutrophils, and two had plasma cells on skin infiltrate. Eosinophils, vasculitis, vasculopathy or signs of chronic venous insufficiency were not observed. Fite-Faraco staining showed intact isolated and grouped acid-fast bacilli in all skin samples (Fig. 2). Histopathology of patient #2 showed infiltrate with predominance of lymphocytes, plasma cells, histiocytes, and necrotic areas, and isolated or grouped intact acid-fast bacilli on Fite-Faraco.

Skin ulcers improved rapidly after multidrug therapy for multibacillary leprosy (MDT-MB) according to WHO recommendations. Some patients started to show partial healing after only 1 week of treatment. Complete healing of the lesions occurred after a mean of 6.9 months (range: 1–12 months) on MDT-MB. No other specific treatment for ulcers was prescribed.

## Discussion and conclusions

Patients with leprosy rarely present ulcerated lesions; these can eventually appear during reactional sates, LP, or secondary to neuropathy, the latter particularly on the plantar surfaces [10]. In general, healing of the ulcerated neuropathic lesions is very slow, due to the vascular and neural disabilities that underlie these manifestations, requiring specific therapeutic interventions in addition to MDT-MB. LP is a rare complication of multibacillary leprosy, due to massive bacilli invasion of endothelial cells causing a thrombotic syndrome, where the initial macular lesion is purpuric. Also, LP usually starts before treatment, and histology shows thrombosis with or without necrosis of small vessels [11]. None of our patients presented clinical or histopathological evidence of LP or reactional states, or a history of ulcers related to trauma. The fast healing of ulcers with MDT-MB alone was a clue to the diagnosis of specific skin ulcer caused by leprosy, which was confirmed by positive bacilloscopy of skin specimens. In type two reaction, skin lesions usually start during leprosy treatment, neutrophils are a hallmark, and acid-fast bacilli, when present, are fragmented [11, 12]; in the two patients with neutrophils on skin specimens, the acid-fast bacilli were intact, and the rapid response to MDT-MB with no treatment for reactional states confirmed the diagnosis of specific skin ulcers caused by leprosy.

Delayed diagnosis or misdiagnosis contributes to advanced disease and irreversible disabilities. Only three of eight patients were diagnosed with less than 6 months since onset of the lesions. Patient #4 had lower limb ulcers for 4 years before the diagnosis of leprosy. He had been treated for venous insufficiency without improvement, had skin ulcers and diffuse infiltration of the skin, alopecia, hypochromic and erythematous macules, and grade two disability, raising the suspicion of leprosy. Patient #2 had skin lesions since 13 years-old, but was diagnosed only 2 years later. At diagnosis, he had multiple infiltrated papules and nodules, some of them ulcerated, and loss of sensation on lower limbs; he was diagnosed as histoid leprosy (Fig. 1i-j). He promptly responded to MDT-MB and was the single patient who did not develop permanent deforming disabilities.

It is not clear why leprosy lesions progressed to ulcer formation. The pathogenesis of skin ulcers is still a subject of intense research, especially of chronic venous leg ulcers [13]. These studies showed that a range of vascular alterations results in a final common path: a chronic and poorly regulated subcutaneous inflammatory process that ultimately causes necrosis and ulceration [14]. In fact, the inflammatory process described in chronic venous ulcers share some features with the inflammatory response of lepromatous leprosy, but not with that of tuberculoid leprosy, where the inflammatory response is well organized, typically with compact granulomas, rarely resulting in necrosis. Similar to lepromatous leprosy, in venous ulcers there is a cellular infiltration with predominance of macrophages, followed by T-lymphocytes, which are important in the events preceding ulceration [15]. As in leprosy, the infiltrating macrophages were associated with production of tumor necrosis factor [16]. In addition, among the main factors generated by the venous ulcer inflammatory process that contribute to necrosis and ulceration are reactive oxygen species [13, 14]. In leprosy, studies have showed that multibacillary, but not paucibacillary patients, had enhanced oxidative stress [17]. Although these findings unveil possible mechanisms for ulceration in lepromatous leprosy lesions, it remains to be established why this occurs in only a subset of patients.

In addition to *M. leprae*, *M. lepromatosis* was also recognized as etiologic agent for leprosy in 2008 [18]. The first reports of these mycobacteria were described in two Mexican patients who died of diffuse lepromatous leprosy. This species was initially associated with diffuse lepromatous leprosy as well as lepromatous leprosy [18]. However, more recently, an analysis of 46 Brazilian patients with leprosy showed that *M. lepromatosis* was present in seven patients, all with tuberculoid leprosy [19]. Unfortunately it was not possible to determine the infecting species in our patients. Whether the infection with *M. lepromatosis* is associated with specific clinical manifestations in Brazil, including development of specific skin ulcers, is not yet known.

This series of cases highlights the importance of recognizing ulcers as a specific cutaneous manifestation of leprosy. Ulcerated lesions should be considered as a clinical manifestation of leprosy, allowing early diagnosis and treatment, and therefore avoiding development of disabilities and persistence of the transmission chain of *M. leprae*.

## Data Availability

The datasets used or analyzed for this study are available from the corresponding author.

## Abbreviations

LP: Lucio’s phenomenon
MDT-MB: multidrug therapy for multibacillary leprosy
